# The Bacterial Mfd Protein Prevents DNA Damage Induced by the Host Nitrogen Immune Response in a NER-Independent but RecBC-Dependent Pathway

**DOI:** 10.1371/journal.pone.0163321

**Published:** 2016-10-06

**Authors:** Claire Darrigo, Elisabeth Guillemet, Rozenn Dervyn, Nalini Ramarao

**Affiliations:** Micalis Institute, INRA, AgroParisTech, Université Paris-Saclay, 78350 Jouy-en-Josas, France; Institut de Pharmacologie et de Biologie Structurale, FRANCE

## Abstract

Production of reactive nitrogen species is an important component of the host immune defence against bacteria. Here, we show that the bacterial protein Mfd (Mutation frequency decline), a highly conserved and ubiquitous bacterial protein involved in DNA repair, confers bacterial resistance to the eukaryotic nitrogen response produced by macrophage cells and during mice infection. In addition, we show that RecBC is also necessary to survive this stress. The inactivation of *recBC* and *mfd* genes is epistatic showing that Mfd follows the RecBC repair pathway to protect the bacteria against the genotoxic effect of nitrite. Surprisingly given the role of Mfd in transcription-coupled repair, UvrA is not necessary to survive the nitrite response. Taken together, our data reveal that during the eukaryotic nitrogen response, Mfd is required to maintain bacterial genome integrity in a NER-independent but RecBC-dependent pathway.

## Introduction

The spore-forming bacterium *Bacillus cereus* is an emerging pathogen and currently the second causative agent of confirmed and suspected food-borne outbreaks (FBO) in France after *Staphylococcus aureus* [1, 2]. Nevertheless, the number of outbreaks is likely underestimated because *B*. *cereus* is rarely sought during FBO investigations [3]. *B*. *cereus* can induce two types of gastrointestinal diseases, leading to emetic or diarrhoeal syndromes. The symptoms associated with *B*. *cereus* are generally mild and self-limiting, but more serious and even fatal cases have been described [4]. *B*. *cereus* can also cause severe local or systemic non-gastrointestinal infections [5–10]. During infection, *B*. *cereus* is able to persist in its host and to resist the host immune system [11–13]. We have previously shown that *B*. *cereus* bacteria are able to escape from macrophages after phagocytosis [14] and to induce cell death by apoptosis [15, 16]. Macrophages are key cells of the host immune response. They produce toxic substances including reactive and nitrogen species. These compounds induce lesions to bacterial DNA, thus limiting bacterial growth within hosts [17, 18]. It has been previously shown that several DNA repair pathways, such as the nucleotide excision repair (NER) pathway and the base excision repair (BER) pathway protect bacteria from the genotoxic effects of the host NO [19, 20]. RecBCD-dependent recombinational repair also plays a role in preventing the genotoxic effects of NO [21]. Damages to DNA that lead to stalling of the RNA polymerase (RNAP) triggers a specialized DNA repair mechanism, called transcription coupled repair (TCR) pathway. Mfd (Mutation Frequency Decline) is an evolutionarily conserved bacterial protein involved in TCR [22]. Mfd has two distinct roles: first it forced the RNAP forward and removes it from the DNA template [22]; second it recruits UvrA to activate the NER pathway [23, 24]. Mfd is implicated in DNA repair and adaptive mutagenesis following artificially induced and spontaneous mutations [25–28]. Mfd was also found to be important for processing the genetic damage during *Bacillus subtilis* sporulation and DNA recombination [29–31]. In addition, Mfd has been implicated in the development of antibiotic resistance in *Campylobacter jejuni* and *H*. *pylori* [32, 33].

In this study, we characterize various phenotype of a Mfd deficient mutant in *B*. *cereus*. We show that Mfd promotes resistance to the deleterious effect of the host nitrogen response. We demonstrate that Mfd prevents bacterial DNA damage in a NER-independent but RecBC-dependent pathway.

## Results

### Phenotypic characterization of the *B*. *cereus* Δ*mfd* mutant

The role of Mfd in DNA repair has been reported mainly for *E*. *coli* and *B*. *subtilis*. However, sofar the analysis of Δ*mfd* mutants did not reveal a defect in bacterial survival, even in mutagenic conditions [34, 35].

**Fig 1:**
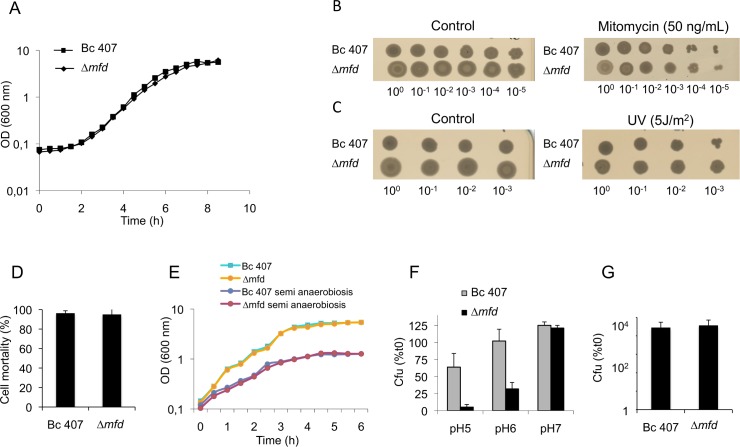
Phenotypical analysis of the Δ*mfd* mutant in mutagenic conditions. (A) *B*. *cereus* wild type and Δ*mfd* mutant strains were inoculated in LB medium at a starting optical density (OD) of 0.07 and grown at 25°C with agitation. The OD was measured every hour at 600 nm. This graph represents representative growth curves out of at least five independent experiments. (B) *B*. *cereus* wild type and Δ*mfd* mutant strains were grown at 37°C under agitation until mid exponential growth phase. Serial dilutions were plated on agar plates containing 50 ng/mL mitomycin C. Plates were incubated ON at 37°C and bacterial survival was assessed by observing the growth zone. Images correspond to a representative example out of at least 3 independent experiments done in duplicates. (C) *B*. *cereus* wild type and Δ*mfd* mutant strains were grown at 37°C under agitation until mid exponential growth phase. Serial dilutions were plated on agar plates and exposed to UV light for 0 to 15 seconds at 5J/m^2^. Plates were incubated ON at 37°C and bacterial survival was assessed by observing the growth zone. Images correspond to a representative example out of at least 3 independent experiments done in duplicates. (D) *B*. *cereus* wild type and Δ*mfd* mutant strains were grown in LB medium until entry into stationary growth phase. Culture supernatant was filtered and added to HeLa cells. Cytotoxicity was measured by the trypan blue method after 2 h of incubation. Results are means of three independent experiments. (E) Bacterial strains were grown in LB medium at 37°C under agitation or without agitation (semi anaerobiosis). This graph represents representative growth curves out of at least three independent experiments. (F) Bacterial strains were grown in LB medium at 37°C under agitation, then diluted with the pH adjusted to 5, 6 or 7. Cfu were calculated after 24 h of growth by plating serial dilutions on LB agar plates. Results are means of at least three independent experiments. (G) *B*. *cereus* wild type and Δ*mfd* mutant strains were cultured in LB medium for 24 h in the presence of the anti microbial peptide cecropin A. Cfu were calculated by plating serial dilutions on LB agar plates. Results are means of three independent experiments.

In our case, the *mfd* mutant strain growth curve was undistinguishable from that of the wild type strain in LB medium (Fig 1A). Addition of mutagenic agents or conditions such as mytomicin C or UV light did not affect the survival of the Δ*mfd* mutant compared to the wild type strain (Fig 1B and 1C). This is consistent with previous reports showing for *E*. *coli* or *B*. *subtilis* Δ*mfd* mutants a high UV mutability despite a minimal UV or mitomycin sensitivity [34, 35]. In *Clostridium difficile*, toxin production is modified in the Δ*mfd* mutant [36]. We assessed whether in our mutant *mfd* deletion could have a role in the induction of cell toxicity as we have previously shown that toxicity is due to the production of extracellular toxins in *B*. *cereus* [15, 37]. As shown in Fig 1D, the wild type and *mfd* mutant strains induced the same toxicity on eukaryotic cells. To mimic *in vivo* conditions, the survival of the strains was assessed in semi anaerobiosis and at acidic pH conditions. As shown in Fig 1E, the *mfd* mutant strain growth curve was indistinguishable from that of the wild type strain in LB medium without agitation (semi anaerobiosis). By contrast, the survival of the *mfd* mutant was severely impaired at acidic pH compared to the wild type strain after 24 h of growth, suggesting that Mfd plays a role in protecting *B*. *cereus* in acidic conditions (Fig 1F).

### Mfd promotes bacterial survival in nitrogen stress conditions

**Fig 2:**
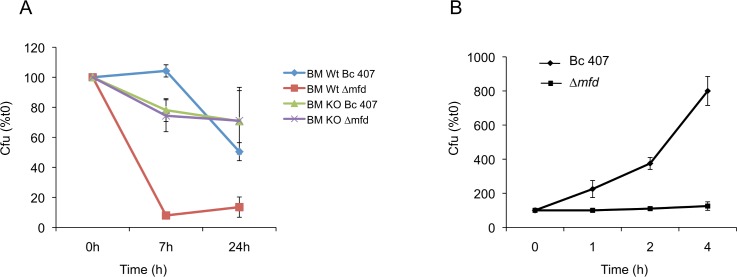
Mfd confers resistance to NO stress. (A) Mice peritoneal macrophages isolated from wild type (BM wt) and NOS2-/- (BM KO) mice were infected with *B*. *cereus* wild type and Δ*mfd* mutant strains. At the indicated time points, bacterial survival was calculated by plating and normalized to the cfu obtained at t0. Results represent mean values of at least 3 independent experiments done in triplicates. (B) Bacteria were exposed directly to chemically generated NO (1.5 mM sodium nitrite) in a cell free system for the indicated times. The bacteria were then harvested and plated on agar plates to evaluate bacterial survival. Results represent mean values of at least 3 independent experiments done in triplicates.

The three major cellular immune resistance mechanisms are synthesis of antimicrobial peptides, production of ROS and NOS. To assess the potential role of antimicrobial peptides on the survival of the strains, the antimicrobial peptide cecropine was added to the growth cultures of wild type and mutant strains, and bacterial survival was assessed by plating serial culture dilutions (Fig 1G). Addition of cecropine had no effect on the growth of the wild type strain as previously described [38]. Similarly, cecropine had no effect on the growth of the Δ*mfd* mutant strain, implying that Mfd did not play a role in the resistance to this antimicrobial peptide. Previous finding have shown that Mfd is not required for transcription recovery following oxidative stress in *E*. *coli* [39]. To gain further insights into the role of Mfd during the host nitrogen response [40], bone marrow (peritoneal) macrophages were isolated from wild type and iNOS-KO mice deficient in NO production (C57/Bl6 nos2-/-). The macrophages were infected with wild type and *Δmfd* mutant strains and bacterial survival was recorded over time. In macrophages derived from wild type mice, the survival of the *Δmfd* mutant strain was severely impaired compared to the survival of the wild type strain (P<0.01 at 7h) (Fig 2A). In contrast, the survival of both the wild type and the *Δmfd* mutant strain were identical in macrophages derived from iNOS-KO mice (P>0.4). These data show that Mfd is essential to survive the deleterious effect of NO produced by phagocytic cells. The bacterial survival for both strains was slightly higher in iNOS-KO mice compared to wild type mice, suggesting that NO affected bacterial survival even in the presence of Mfd.

To confirm the role of Mfd in bacterial resistance to NO stress, bacteria were exposed directly to chemically generated NO in a cell free system (Fig 2B). In this system, the growth capacity of the *mfd* mutant was strongly impaired compared to that of the wild type (P<0.007 at 4 h). This demonstrates the implication of Mfd in the resistance to NO, a critical mediator of the host innate immune response. The effect was seen using chemical sources of NO and human phagocytic cells-derived NO, highlighting the direct biological relevance of our finding.

### Mfd promotes bacterial survival *in vivo* in the context of NO stress

Wild type and iNOS-KO mice were inoculated intranasally with wild type and Δ*mfd* mutant bacteria. After 3 days post infection, the mortality of wild type mice infected with Bc 407 reached 70% (7/10). By contrast, mortality of wild type mice infected with the Δ*mfd* mutant only reached 44% (4/9). In iNOS-KO mice, mortality was of 71% (5/7) for mice infected with Bc 407 and 75% (6/8) for iNOS-KO mice infected with the Δ*mfd* strain. No additional death occurred after 3 days post infection. These data demonstrate the strong implication of Mfd in the resistance to the host NO response *in vivo*.

**Fig 3:**
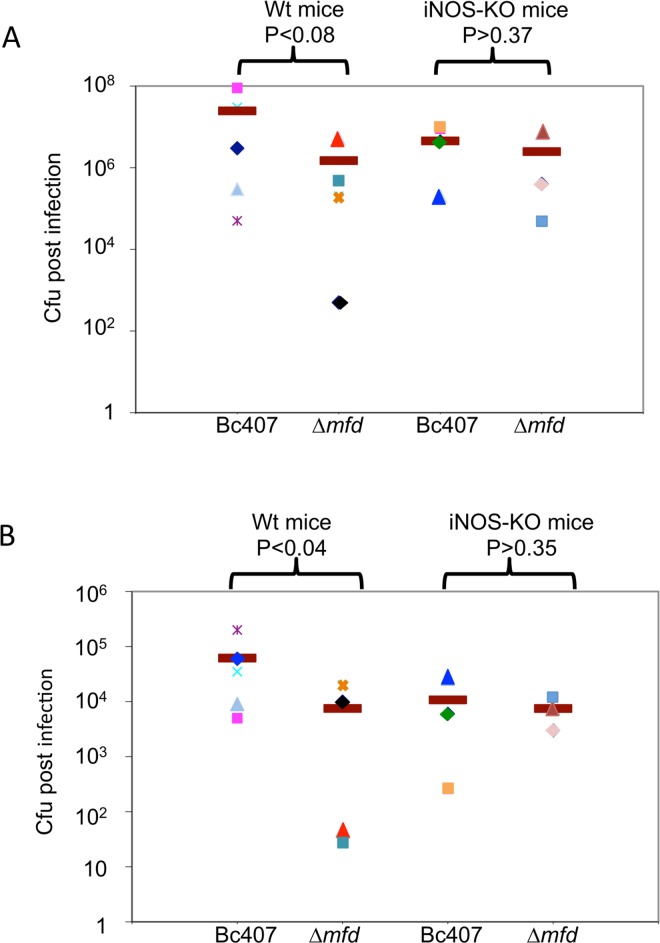
Role of Mfd in *in vivo* survival following NO stress. C57/Bl6/Sev 129 mice (Wt Mice) and NOS2-/- mice (iNOS-KO mice) were inoculated intranasally with *B*. *cereus* wild type and Δ*mfd* mutant bacteria (5.10^6^/mice). Wild type and *Δmfd* mutant strains were recovered from the lung (A) and from the brain (B) following mice death. The cfu recovered post infection was calculated by plating the bacteria on LB agar plates. For each mouse, the same symbol is used for lung (A) and brain (B) values. Cfu is shown for 5 wild type mice infected with Bc407, 4 wild type mice infected with Δ*mfd*, 3 KO mice infected with Bc407 and 3 KO mice infected with Δ*mfd*.

To complete these data, wild type and *Δmfd* mutant strains were recovered from various organs following infection. The lungs and brain of the mice were collected and crushed after animal death or at day 7 were the surviving mice were euthanized. The bacterial cfu was calculated by plating serial dilutions on LB agar plates. In euthanized survivor animals, no bacteria were recovered (not shown). By contrast, it is noteworthy that in dead animals, bacteria were recovered from various organs showing that *B*. *cereus* is able to disseminate from the nose to the lung and the brain (Fig 3A and 3B) and to a lower extent to the liver and spleen (no shown). Interestingly, it seems that within the same mice, the cfu recovered from the lung was inversely correlated to the cfu recovered from the brain, suggesting that the bacteria may colonize preferentially either the brain or the lung. In average, the amount of the Δ*mfd* mutant cfu recovered in the lung and the brain after infection of the wild type mice was lower than the cfu recovered for the wild type strain (P<0.08 in the lung and P<0.04 in the brain). This indicates that Mfd promotes bacterial survival *in vivo*.

In average, fewer wild type bacteria were recovered from the iNOS-KO mice compared to wild type mice, despite a similar mortality (71% and 70%), suggesting an increased sensitivity of these mice to infection. In addition, the cfu obtained from the wild type strain in the lung and brain of iNOS KO mice were not statistically different from the cfu obtained for the Δ*mfd* mutant, implying that in the absence of NO, Mfd was not necessary to provide bacterial survival (P>0.37 in the lung and P>0.35 in the brain). In the iNOS-KO mice, the cfu of the wild type and Δ*mfd* mutant strains was similar and lower than the cfu obtained from the wild type strain in wild type mice showing the higher sensitivity of the iNOS-KO mice, which reached similar mortality rates with both strains (71% for the wild type and 75% for Δ*mfd*). This may explain why the average amount of cfu of the Δ*mfd* strain was similar in wild type and iNOS-KO mice, despite a strong difference in mice mortality (44% mortality for the wild type mice and 75% mortality for the iNOS-KO mice).

### RecBC (AddAB) but not UvrA is involved to prevent NO stress

**Fig 4:**
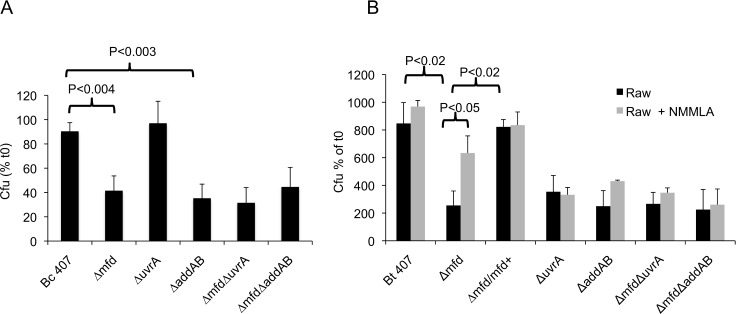
**(A) AddAB but not UvrA is involved to prevent NO stress**. *B*. *cereus* wild type and mutant strains were exposed to 1.5 mM NO for 1 h in a cell-free system. Bacteria were harvested and plated on agar plates to evaluate bacterial survival. Cfu counts were normalized to initial cfu. The results reported are mean values of at least three independent experiments. P values are calculated using the Student test. **(B) Survival of *B*. *cereus* mutant strains with Raw cells.** Raw cells were infected with *B*. *cereus* wild type and mutant strains at a multiplicity of infection of 10 at 37°C. After 20 h of incubation, bacteria were recovered by scraping and counted by plating serial dilutions on agar plates. Alternatively, the NO inhibitor NMMLA was added to the cells at 1 mM.

In *E*. *coli*, when the RNAP is stalled on a DNA lesion, Mfd contributes to the TCR pathway by recruiting UvrA. We thus investigated the role of UvrA during NO damage. Surprisingly, resistance to NO stress was not impaired in the Δ*uvrA* mutant (Fig 4, Table 1). Deletion of *uvrA* in a Δ*mfd* mutant had no effect as the Δ*mfd*Δ*uvrA* double mutant had a phenotype similar to the Δ*mfd* single mutant.

**Table 1:** NO IC 50 on *B*. *cereus* mutants.

	IC 50 (mM NO)	Error to the mean
Bc 407	4.12	1.2
Δmfd	1.61	1.3
ΔuvrA	3.92	1.2
ΔaddAB	1.06	1.3
ΔmfdΔuvrA	1.49	1.3
ΔmfdΔaddAB	1.32	1.4

*B*. *cereus* wild type and Δ*mfd*, Δ*uvrA*, Δ*addAB*, Δ*mfd*Δ*uvra*, Δ*mfd*Δ*addAB* mutant strains grown until entry into mid exponential growth phase were incubated at 37°C for 1 h with 0, 0.5, 1.5, 2.5, 3.5, 5 and 10 mM of NO. Bacterial survival was recorded by plating serial dilution on agar plates. The NO concentration required to decrease by 50% the survival rate of each strain during NO stress (IC 50) was calculated using the GraphPad PRISM software (version 6.0, GraphPad Software, San Diego, CA) with non-linear regression. Data are means of at least 5 independent experiments.

It has been previously shown that RecBC plays a role in preventing NO damage. We therefore investigated the role of RecBCD during NO stress with Mfd deficiency/proficiency (Fig 4, Table 1). In *B*. *cereus* the homologue of *E*. *coli* RecBC is AddAB. We found that resistance to NO stress was impaired in the *B*. *cereus* Δ*addAB* mutant compared to the wild type strain (P<0.003). The double mutant Δ*mfd*Δ*addAB* was similar to the Δ*mfd* or Δ*addAB* single mutants suggesting that Mfd and AddAB act in the same repair pathway.

Taken together, our data show that in the case of NO-induced lesions, Mfd promotes DNA repair without UvrA or NER contribution, but by following the RecBC-dependent pathway.

To test the role of Mfd, UvrA and AddAB during resistance to NO produced by cells, the survival rates of the wild-type and mutant strains were assessed after incubation of the Raw macrophage-like cell line in the presence of an inhibitor of the nitrogen response, N^G^-monomethyl-L-arginine (NMMLA), a competitor of the iNOS enzyme (Fig 4B). The survival of the Δ*mfd* mutant was impaired following incubation of cells in the absence of NMMLA compared to the wild type strain (P<0.02). By contrast, in the presence of the inhibitor, the survival rate of the *Δmfd* increased significantly, further demonstrated the role of Mfd in resistance to NO stress. The survival rate of Δ*mfd* mutant with NMMLA only partially reached the survival of the wild type strain suggesting that Mfd may play a role in protecting the bacteria against other cellular stresses. We have consistently shown that Mfd is also important for survival at acidic pH. Alternatively, the partial effect of NMMLA could reflect an incomplete inhibition of the NO production. The complemented Δ*mfd/mfd*+ strain shows a survival rate comparable to the wild type strain. The survival of the Δ*uvrA* and Δ*addAB* mutants was also impaired in the cells, suggesting that these factors are essential to promote bacterial survival in cells. However, addition of NMMLA did not increase the mutant survival. This suggests that UvrA and AddAB are also essential to survive to other stresses than NO. This is consistent with previous findings showing the implication of UvrA and RecBC in the resistance to reactive oxygen species (ROS) [41, 42].

## Discussion

### Mfd confers *B*. *cereus* resistance to the host immune NO response

We analysed several phenotypes of the *B*. *cereus* Δ*mfd* mutant. Mfd does not play a significant role in protecting the bacteria against mitomycin or UV light. By contrast, Mfd allows bacterial survival in the context of stresses that may promote their resistance to the bactericidal activities of macrophages, such as acidic pH and the host nitrogen response. To gain additional insights into the role of Mfd during bacterial resistance to NO, we show that Mfd allows bacterial survival in the context of NO stress produced by bone marrow macrophages. *In vivo*, Mfd allows bacterial survival following the host immune nitrogen response. Thus, our results reveal a new role of Mfd during bacterial pathogenesis. Mfd is well conserved among bacterial species, so these observations reveal a novel mechanism that may be used by a large spectrum of bacteria to counteract the host immune system, and in particular the mutagenic properties of reactive nitrogen species, an important mutagenic component of the host immune defence against bacteria.

### Mfd prevents bacterial DNA damage in a NER-independent pathway

In *E*. *coli*, Mfd was previously shown to have two distinct specific roles: i) Mfd removes RNAP blocked on DNA and ii) Mfd recruits UvrA to promote lesion repair by NER [27]. These two mechanisms are not necessarily coupled. Indeed, depending on the nature of DNA damage or the reason of RNAP stalling, Mfd can promote the removal or the forward translocation of arrested RNAP in the absence of repair and/or independently of UvrA recruitment [26, 43]. For example, Mfd can release RNAP and the truncated transcript from a transcriptional roadblock caused by a DNA-bound protein [44], sometimes causing transcription termination, without UvrA recruitment [45]. In the case of NO-induced damage, our results reveal that the implication of Mfd is independent of UvrA. One possibility is that the NER-independent protective action of Mfd is to prevent sDNA breaks associated with RNAP backtracking. Indeed, it has been previously shown that codirectional collisions with backtracked elongation complexes often result in double strand breaks (DSBs) and that Mfd retrieve codirectional DNA damage by releasing arrested elongation complexes [46]. Another hypothesis may be that Mfd normally uses UvrA but that without it, an alternative mechanism is used, hiding the phenotype in the Δ*uvrA* mutant.

### Mfd confers resistance to NO via RecBC/AddAB

**Fig 5:**
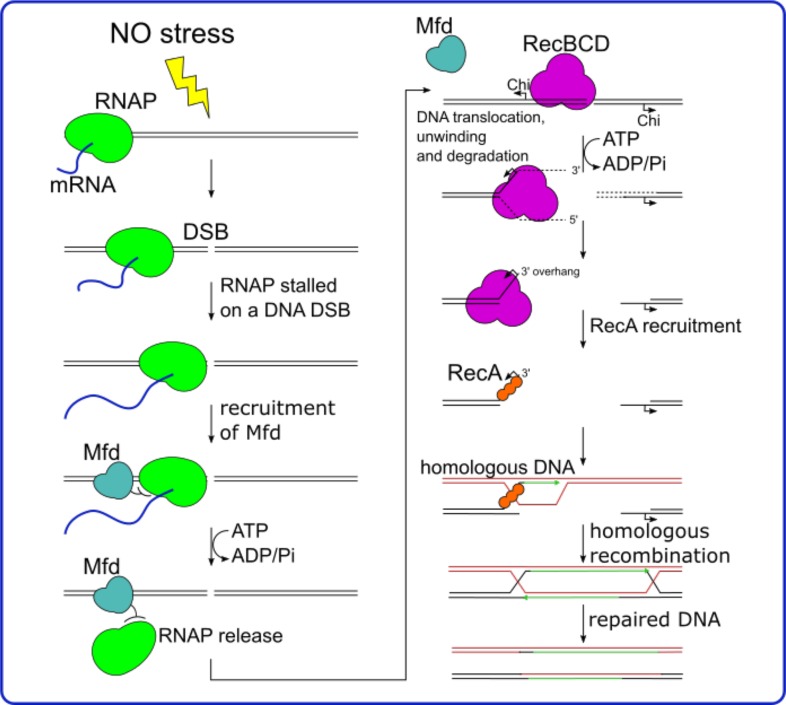
In the context of NO stress, we show that *mfd* and *recBC*/*addAB* deletions are epistatic. As RecBC participates in the repair of Double Strand Breaks (DSB), this indicates that Mfd also participates in DSB repair. It is unlikely that Mfd prevents DSBs because, in that case, RecBC would be essential for DNA repair in a *mfd* mutant. By contrast, we hypothesized that following the DSBs induced by NO exposure, RecBC has not directly access to Double strand ends (DSEs). Thus, Mfd would act first by removing the RNAP blocked on DNA lesions. Then the RecBCD complex can be recruited to repairs the DSB. RecBCD unwinds the DNA helix and degrades single strands. When RecBDC encounters a Chi site on the DNA, the degradation of the 5’ terminus is enhanced leaving a 3’ overhang and leads to the formation of an ssDNA. RecA binds to ssDNA and promotes repair by recombination with a homologous molecule of DNA [50].

We show here that Mfd is required for NO resistance in *B*. *cereus*. Resistance to NO stress was also impaired in the *B*. *cereus* Δ*addAB* mutant. This is in agreement with previous findings showing the implication of RecBC in NO resistance of *Salmonella enterica* and *E*. *coli* [21, 47]. The role of RecBC (AddAB) during NO response suggests that NO-induced DNA damages are not target for NER but rather require a common pathway to Mfd and RecBC to repair DNA fragmentation. The NO sensitivity in the absence of RecBC-dependent homologous recombination indicates that NO toxicity is due, at least partially, to the formation of DNA double-strand breaks (DSBs) [21]. If Mfd played a role in preventing DSBs, linear DNA would not accumulate in the *mfd* mutant provided that DSB repair is functional, and RecBC would be essential for DNA repair in a *mfd* mutant. In contrast, *mfd* and *recBC*/*addAB* mutations are epistatic, which indicates that Mfd participates in DSB repair, a new role for this protein. RecBC act first during DSB repair by binding to the DNA double-strand end (DSE), therefore the only way Mfd could act before RecBC would be if DSEs are not directly available to RecBC after NO damage. It is conceivable that Mfd removes the RNAP arrested by a DNA lesion, or repair protein complexes attached to DSEs in NO treated cells, to allow RecBC action (Fig 5). Interestingly, Mfd has also been hinted to contribute to homologous recombination in *B*. *subtilis*, and this may help during DNA repair [31].

Previous findings on *Salmonella typhimurium* showed that NO induces DNA damage targeted by the Base Excision Repair (BER) pathway [20]. BER is involved in the recognition of modified bases by specific DNA glycosylases, which action on NO-induced DNA damage can induce homologous recombination [48]. As the formation of DSBs is usually linked to the activation of RecBC-dependent homologous recombination [49], it would be interesting to study the link between Mfd, RecBC and BER following NO genotoxic bacterial DNA damage.

Taken together, our findings demonstrate an important role of the DNA repair enzyme Mfd during bacterial resistance to NO stress, by limiting the genotoxic effects of NO generated by the host inflammatory response. These findings allow better defining the mechanistic pathway of bacterial resistance to NO and the efficient contribution of Mfd, a well-conserved bacterial protein, in bacterial survival during pathogenesis.

## Material and Methods

### Bacterial strain and mutant construction

The *B*. *thuringiensis* strain 407 Cry—(Bc 407) was used as a model for *B*. *cereus*. This strain has been cured of its plasmid, is acrystalliferous, and shows high phylogenic and phenotypic similarity with the *B*. *cereus* reference strain ATCC 14579 and is therefore considered as a *B*. *cereus* strain [51].

The Bc 407 *Δmfd* mutant was constructed as follows. The *mfd* gene was disrupted through double homologous recombination using the thermosensitive vector pMAD. *Bam*HI-*Xba*I (515 bp) and *Pst*I-*EcoR*I (512 bp) DNA fragments corresponding to upstream and downstream regions of the *mfd* gene were generated from the Bc 407 chromosome by PCR using the primer pairs:

mfd-1 (5’-CGCGGATCCGTAGGCTCCATTAACGCAG-3’),
mfd-2 (5’-GCTCTAGACACCAAGTAACGCCACTAAATC-3’), and
mfd-3 (5’-AACTGCAGGAGGTGTTTCTGCAATTGAGG-3’),
mfd-4 (5’-CCGGAATTCGCTGCCTCATTTCTACTG -3’).

A Kan^R^ cassette carrying a *kan* gene was purified from pDG783 [52] as a 1.6 Kb *Xba*I-*Pst*I fragment. The amplified DNA fragments and the Kan^R^ cassette were digested with the appropriate enzymes and assembled together by ligation to produce a “*mfd*-upstream”-“Kan^R^ cassette”-“*mfd*-downstream” *Bam*HI-*EcoR*I fragment, which was then inserted between the *Bam*HI and *EcoR*I sites of pMAD. The resulting plasmid was introduced into Bc 407 by electroporation [53] and the *mfd* gene was deleted by a double crossover event as previously described [54]. Chromosomal allele exchange was confirmed by PCR with oligonucleotide primers located upstream from mfd-1, mfd-5 (5’-AACTGCAGGCAGACACTGCGGAGG -3’) and downstream from mfd-4, mfd-6 (5’-TGCTCTAGACCTTCGGGATTACTACCCTGCC-3’), and in the Kan^R^ cassette (5’-CGGGTCGGTAATTGGGTTTG-3’), (5’-GCAGCTGCACCAGCCCCTTG-3’). The insertion mutant strain was designated Bc 407 Δ*mfd*.

To complement the Bc 407 *Δmfd* mutant, the *mfd* gene, including the coding sequence and the promoter region (as defined in http://subtiwiki.uni-goettingen.de/wiki/index.php), was obtained using the primer pair mfd-5 and mfd-6, and the sequence of the obtained fragment was verified. The pHT315 cloning vector [53] containing the complete *mfd* gene and its promoter was used to transform Bc 407 Δ*mfd* strain by electroporation. Transformants were selected for resistance to erythromycin. The resulting new strain was designated Bc 407 Δ*mfd* / *mfd*^*+*^.

The Bc 407 Δ*uvrA* and *ΔmfdΔuvrA* mutants were constructed as follows. A *Nhe*I-*sph*I (936 bp) and *Eco*RI-*Bam*HI (1006 bp) DNA fragments corresponding to upstream and downstream regions of the *uvrA* gene were generated from the Bc407 chromosome by PCR using the primer pairs:

uvrAB-F1 (5’- CTAGCTAGCCGCAACAATCGCTACACCAA -3’),
uvrAB–R1 (5’- CCGCGCATGCAATAAGTTTATTGATGAAAACCGAG -3’), and
uvrAB–F2 (5’- CCGGAATTCCAAGTATAATCCGACCTCCTCA -3’),
uvrAB–R2 (5’- CGCGGATCCTACGGGAAAAAGGATTTGAAGAAG -3’).

A Tet^R^ cassette carrying a *tet* gene was purified from pHTS1 [52] as a 1.6 Kb (*Sph*I and *Eco*RI) fragment. The amplified DNA fragments were digested with the appropriate enzymes and inserted between the *Nhe*I and *Bam*HI sites of pRN5101 [55]. The resulting plasmid was introduced into Bc407 and Δ*mfd* strains by electroporation and the *uvrA* gene was deleted by a double crossover event. Chromosomal allele exchange was confirmed by PCR with oligonucleotide primers *uvrA* forw (5’- TTTTACTGTATGCAGTTTTCTACGGG -3’) and *uvrA rev* (5’- GAGGTTCCACAATTGCCTTCTC -3’), and in the Tet^R^ cassette (5’-CGGGTCGGTAATTGGGTTTG-3’), (5’-GCAGCTGCACCAGCCCCTTG-3’). The insertion mutant strains were designated Bc 407 Δ*uvrA* and Bc 407 Δ*mfd*Δ*uvrA*.

The Bc 407 Δ*addAB* and *ΔmfdΔaddAB* mutants were constructed as follows. A DNA fragment corresponding to an internal region of the *addAB* gene (1000 bp) was generated from the Bc 407 chromosome by PCR using the primer pairs addAB-1 (5’- CGGGATCCCGCGGTGATGATATGGGTACGGCG -3’) and addAB-2 (5’- CGGCTAGCCGCGATTTGTTCTTGTAGCGTTTCGGCCG -3’). This DNA fragment was inserted between the *Bam*H1 and *Nhe*I sites of pRN5101, and the resulting plasmid was introduced into Bc 407 and Bc 407 Δ*mfd* by electroporation. Integration of the recombinant plasmid was confirmed by PCR using primers mapping to the ends of pRN5101 and primers external to the insertion site. The stability of the gene disruption was tested by growing the bacteria in LB medium for 15 generations, plating onto LB agar plates and re-picking the colonies onto LB agar plates containing 5 μg/mL erythromycin. The calculated mutant stability was >98%. The insertion mutant strains were designated Bc407 Δ*addAB* and Bc407 Δ*mfdΔaddAB*.

### Bacterial growth conditions

For unstressed conditions, all strains were cultivated in LB medium with agitation at 25°C or 37°C.

For phenotypic studies, various stress conditions were established as follows. Bacterial strains were grown in LB medium with shaking at 37°C until entry into exponential growth phase. The culture was divided in two. One of the resulting half-cultures was incubated at 37°C (control), whereas the other half was grown without agitation (semi anaerobiosis). The optical density was measured at 600 nm over time. Alternatively, bacteria were grown in LB medium until mid exponential growth phase at 37°C with shaking. Cultures were then diluted to 10^4^ cfu/ml in RPMI-1640 medium (Invitrogen) adjusted at various pH (5, 6 and 7) and bacterial survival was assessed by plating after 24 h.

### Resistance to cecropin A

*B*. *cereus* wild type and *mfd* mutant strains were cultured in LB medium for 24 h in the presence of the anti microbial peptide cecropin A (Sigma) [38]. The remaining cfu was calculated by plating serial dilutions on LB agar plates.

### Cell culture, infection and toxicity

HeLa epithelial cells (provided by ATCC, ATCC® CCL-2^TM^) and Raw 264.7 macrophage cells (provided by ATCC, ATCC® TIB-71^TM^) were maintained in Dulbeco’s modified Eagle’s minimum essential medium (DMEM, Invitrogen) supplemented with 10% FBS. The cells were incubated at 37°C under a 5% CO_2_ atmosphere and saturating humidity. The day before use, cells were detached using 0.02% trypsin, counted with a hematocytometer and seeded into multiwell disposable trays at a density of 2.10^5^ cells per well. Cytotoxity to HeLa cells was assessed as previously described. Briefly, *B*. *cereus* wild type and Δ*mfd* mutant strains were grown in LB medium until entry into stationary growth phase. Culture supernatants were filtered and added to HeLa cells. Cytotoxicity was measured after 2 h of incubation by the trypan blue method [37, 56, 57]. For bacterial survival assays, Raw cells were infected with *B*. *cereus* wild type and Δ*mfd*, Δ*mfd/mfd*+, Δ*uvrA*, Δ*addAB*, Δ*mfd*Δ*uvrA* and Δ*mfd*Δ*addAB* strains bacteria at a multiplicity of infection of 10 at 37°C. After 20 h bacteria and cells were removed from the flask by scraping and bacteria were counted by plating serial dilutions on agar plates. To inhibit the nitrogen response in cellular assay, the NO inhibitor NMMLA N^G^-monomethyl-L-arginine (Sigma) was added to the cells at 1 mM. NMMLA competes with L-arginine for the site of action of the iNOS enzyme.

### UV and mitomycin treatment

*B*. *cereus* wild type and mutant strains were grown at 37°C under agitation until mid exponential growth phase and diluted to 5. 10^7^ cfu/ml. Serial dilutions were plated on agar plates and exposed to UV light (5J/m2) for 0 to 15 seconds. Alternatively, serial dilutions were plated on agar plates containing 50 ng/mL mitomycin C. Plates were incubated ON at 37°C and bacterial survival was assessed by observing the size of the growth zone.

### NO inducer *in vitro*

Bacteria were exposed directly to chemically generated NO+ in a cell-free system as described by Miyagi *et al*. [58] with modifications. Briefly, bacteria were grown in LB medium until mid exponential growth phase at 37°C with shaking. Cultures were then diluted to 10^4^ cfu/ml in RPMI-1640 medium (Invitrogen) in the presence of 0 to 10 mM sodium nitrite (Walco Pure Chemical Industries Ltd); NO+ is generated from the sodium nitrite by a chemical reaction [58]. The bacteria were then cultured at 37°C for 0 to 4 h, harvested and plated on agar plates to evaluate bacterial survival. Using this cell free assay, the NO concentration required to decrease by 50% the survival rate of each strain (IC_50_) was calculated using the GraphPad PRISM software (version 6.0, GraphPad Software, San Diego, CA) with non-linear regression.

### Mice experiments

6 to 8 weeks old C57/Bl6/Sev 129 mice were used for infections. Wild type mice were obtained from breading “Elevage Janvier, France”, and NOS2-/- (iNOS-KO) mice were kindly supplied by Jean Claude Jeanny, Institut des Cordeliers, animalerie centrale de la faculté de Pharmacie de Paris (health monitoring report from Harlan UK Technical service, report reference 07–2948). All protocols and mice experiments were performed and validated by the animal facilities INRA-UIERP in Jouy en Josas according to the ethical rules provided by the 2010/63/UE legislation and approved by the INRA-UIERP in Jouy en Josas Committee on the ethics of animal experiments, named “Comité d’Ethique en Expérimentation Animale du Centre INRA de Jouy en Josas et AgroParisTec » (Comethea, N° SIRET INRA Jouy en Josas: 18 0070039 00078, agreement number B78-720). All experiments were performed under sodium pentobarbital anaesthesia and all efforts were made to minimize animal suffering. 7 to 10 mice were inoculated intranasally with wild type and mutant bacteria (5.10^6^ *B*. *cereus*/mice). Precisely, 10 wild type mice were infected with Bc 407 strain and 9 wild type mice were infected with the Δ*mfd* mutant. 7 iNOS KO mice were infected with Bc407 strain and 8 iNOS KO mice were infected with the Δ*mfd* mutant. Survival was recorded over 7 days. At the time of death of the animals, or at day 7 for survivors, the lungs and brain of the mice were collected, weighted and crushed. The bacterial cfu was calculated by plating serial dilutions on LB agar plates supplemented or not with the appropriate antibiotics.

Animal health was checked daily: general state and behavior, motility, potential bleeding. 1 animal became severely ill after 3 days and showed obvious signs of suffering (decreased motility, shaking, distress, abnormal position), and was sacrificed to avoid suffering. For the other animals, death occurred after a maximum of 3 days. The experiments were stopped at 7 days and the survivors were euthanized at the endpoint. All animals were euthanized by cervical dislocation.

Bone marrow macrophages were obtained from wild type and iNOS-KO mice as previously described [59]. Briefly, Bone-marrow-cells were obtained by flushing the mice femurs and differentiated in culture in RPMI 1640 medium supplemented with 10% FBS and 10% L929 cell-conditioned medium as a source of macrophage colony stimulating factor. Cells were infected with bacteria at a multiplicity of infection of 10 at 37°C. After 0, 7 and 24 h bacteria and cells were detached by scraping and bacteria were counted by serial dilutions on plates.
